# Opioid-free versus opioid-containing labor epidural analgesia: interpreting non-inferiority claims in a retrospective cohort study

**DOI:** 10.1016/j.bjane.2026.844816

**Published:** 2026-08-14

**Authors:** Pourya Baharlooei

**Affiliations:** Tehran University of Medical Sciences, School of Medicine, Tehran, Iran

*Dear Editor*,

I read with interest the 2026 article titled “The Impact of Opioid-Free Labor Epidural Analgesia on Maternal and Infant Outcomes: A Retrospective Cohort Study” by Brahmbhatt et al., examining the impact of Opioid-Free Labor Epidural Analgesia (OFLEA) versus conventional Opioid Epidural Labor Analgesia (OLEA) on maternal and neonatal outcomes.^1^ The authors address a timely and clinically important question. While the findings are promising, there appear to be methodological concerns relevant to interpretation of the results.

The methodology does not adequately support a formal non-inferiority claim. No non-inferiority margin was pre-specified, and the post-hoc analysis across three candidate thresholds (0.75, 1.0, and 1.5 pain units) is more appropriately characterized as exploratory rather than confirmatory. At the midpoint margin of 1.0 pain units, power is reported at 19.7%, limiting the strength of a non-inferiority conclusion. In the absence of an a priori margin, the results are more appropriately understood as exploratory comparative effectiveness evidence.

Future prospective comparative studies should define a non-inferiority margin before data collection begins. Where such studies take the form of randomized controlled trials, adherence to the CONSORT extension for non-inferiority and equivalence designs would further support transparent and interpretable reporting.^2^

The original report describes 1:1 matching by maternal age; the enrolled groups nonetheless differed substantially in size (OLEA: n = 1,093 vs. OFLEA: n = 330). While unequal group sizes alone do not establish baseline imbalance or non-random allocation, limiting the matching strategy to a single covariate is a meaningful constraint. Group-specific baseline characteristics and post-matching balance diagnostics were not clearly reported, leaving it uncertain whether important obstetric and clinical factors were adequately balanced between groups. Future studies should employ propensity score matching or multivariable adjustment for a broader set of clinical covariates and provide transparent reporting of the matching strategy and the balance achieved.

A further consideration concerns the pharmacological composition of the two epidural regimens. The opioid-free group received 0.2% ropivacaine, whereas the opioid-containing group received 0.1% ropivacaine plus fentanyl. The two strategies therefore differed in both opioid content and local anesthetic concentration. Observed differences in pain outcomes cannot be attributed specifically to opioid avoidance alone; the comparison is more accurately understood as one between two pharmacologically distinct epidural treatment strategies, rather than a direct opioid versus no-opioid comparison.

Additionally, the maximum pain score showed a statistically significant difference in the unadjusted Wilcoxon rank-sum test (p = 0.035); however, the corresponding linear regression model adjusted for age, ethnicity, and fever yielded a non-significant result (p = 0.088, 95% Confidence Interval crossing zero). The adjusted estimate is imprecise and compatible with no meaningful between-group difference on this measure. This outcome should be clearly distinguished from those endpoints that retained statistical significance after covariate adjustment. Authors are encouraged to differentiate outcomes that survive adjustment from those that do not, and to avoid characterizing analgesic benefit as established across all pain metrics when adjusted estimates for key endpoints do not support that conclusion.^3^

These limitations do not lessen the importance of the research question, but they do affect the strength of the conclusions. The observed outcomes are encouraging and warrant prospective validation. However, the field would be best served by prospective, adequately powered, multicenter randomized controlled trials with a pre-registered non-inferiority margin, robust covariate adjustment, and standardized outcome reporting. Such studies would provide the evidentiary foundation necessary to advocate for OFLEA as a standard-of-care alternative.

## Data availability statement

No new data were created or analyzed in this article. Data sharing is not applicable to this article.

## Conflicts of interest

The authors declare no conflicts of interest.
